# Linezolid Resistance Genes and Mutations among Linezolid-Susceptible *Enterococcus* spp.—A Loose Cannon?

**DOI:** 10.3390/antibiotics13010101

**Published:** 2024-01-19

**Authors:** Jennifer K. Bender, Carola Fleige, Finn Funk, Clara Moretó-Castellsagué, Martin A. Fischer, Guido Werner

**Affiliations:** 1 Nosocomial Pathogens and Antibiotic Resistances Unit, Department of Infectious Diseases, Robert Koch Institute, 38855 Wernigerode, Germanyfunk.finn@gmail.com (F.F.); mmoreto@bellvitgehospital.cat (C.M.-C.); fischerm@rki.de (M.A.F.); wernerg@rki.de (G.W.); 2 Department of Microbiology and Parasitology, University Hospital of Bellvitge, 08907 Barcelona, Spain

**Keywords:** linezolid resistance, *Enterococcus* spp., silent resistance genes, genotype–phenotype correlation

## Abstract

The National Reference Centre for Enterococci receives an increasing number of linezolid-resistant *Enterococcus* isolates. Linezolid (LIN) resistance is mediated by G2576T 23S rDNA gene mutations and/or acquisition of resistance genes (*cfr, optrA, poxtA*). There are anecdotal reports that those resistance traits may be present in phenotypically linezolid-susceptible isolates. We aimed to determine the prevalence of LIN resistance genes and mutations in enterococci with a LIN MIC of 4 mg/L in broth microdilution (EUCAST = susceptible) isolated from German hospital patients 2019–2021. LIN MICs were additionally determined by ETEST^®^ and VITEK2. Selected strains were subjected to LIN selective pressure and growth was monitored with increasing antibiotic concentrations. We received 195 isolates (LIN MIC = 4 mg/L). In total, 78/195 (40%) isolates contained either a putative resistance gene, the G2576T mutation, or a combination thereof. Very major error was high for broth microdilution. The ability to predict phenotypic resistance from genotypic profile was highest for G2576T-mediated resistance. Selection experiments revealed that, in particular, *E. faecium* isolates with resistance gene mutations or *poxtA* rapidly adapt to MICs above the clinical breakpoint. In conclusion, LIN resistance genes and mutations can be observed in phenotypically linezolid-susceptible enterococci. Those isolates may rapidly develop resistance under LIN selective pressure potentially leading to treatment failure.

## 1. Introduction

Enterococci are gut commensal organisms that might become life-threatening bacterial pathogens in immunocompromised individuals, mainly in hospital settings. Infections are difficult to treat due to their inherent insusceptibility to various antimicrobial substances. Since the year 2000, linezolid (LIN), a synthetic antibiotic, has been approved as a last-resort therapeutic option to treat vancomycin-resistant *Enterococcus* spp. (VRE). Although an increase in the prevalence of linezolid-resistant enterococci (LRE) has not yet been reported on a global scale [1,2,3,4], recent data from the German Antimicrobial Resistance Surveillance indicate an increase of LRE among invasive isolates (0.6% in 2019 to 1.2% in 2021; https://ars.rki.de/, accessed on 26 November 2023). Moreover, and although biased, the National Reference Centre for Staphylococci and Enterococci (NRC) at the Robert Koch Institute has identified an increasing number of LRE sent to the NRC for confirmatory resistance diagnosis [5].

In enterococci, LIN phenotypic resistance is either mediated by the expression of mobile resistance genes *poxtA* or *optrA*, both encoding ribosomal protection proteins, by the methyltransferase Cfr and variants thereof, or the result of chromosomal alterations mainly at position 2576 in the V domain of the 23S ribosomal RNA (reviewed by [6,7]).

In order to determine LIN resistance, antimicrobial susceptibility testing (AST) is performed by broth microdilution (BMD) (reference method), gradient strip tests (such as ETEST^®^), automated systems (such as VITEK2), or disk diffusion assays. According to the European Committee on Antimicrobial Susceptibility Testing (EUCAST), LIN resistance in *Enterococcus* spp. is defined as a minimal inhibitory concentration (MIC) of >4 mg/L. It has previously been demonstrated that enterococci harboring the resistance gene *cfr/cfr(B)*, *optrA*, or G2576T mutations in low allelic frequencies would present with LIN MICs of 4 mg/L (or even below), thus being inadequately diagnosed as linezolid-susceptible isolates (EUCAST breakpoints) [8,9,10,11].

From 2019 to 2021, the NRC noted quite a substantial number of isolates demonstrating an MIC just below the EUCAST-defined clinical breakpoint. Thus, we compiled a strain collection of phenotypically linezolid-susceptible (EUCAST) or linezolid-intermediate (according to the Clinical and Laboratory Standards Institute (CLSI)) *Enterococcus* spp. (LSE) isolates (all MIC = 4 mg/L) as determined by the reference method BMD and aimed at: (i) determining the prevalence of LIN resistance genes and mutations in enterococci with a LIN MIC of 4 mg/L, (ii) comparing the performance of the reference method BMD with the results obtained from VITEK2, ETEST^®,^ and CHROMagar^TM^ LIN-R; (iii) analyzing the genotype–phenotype correlation between the presence of LIN resistance genes/mutations and LIN resistance, and (iv) evaluating the potential of “silent” resistance genes and/or G2576T ribosomal RNA gene mutations in supporting rapid adaption to LIN under antibiotic selective pressure.

Our study revealed a high proportion of LIN resistance genes and/or chromosomal mutations in phenotypically susceptible (according to EUCAST) or intermediate (CLSI) isolates that could potentially result in resistance development under LIN selective pressure. We further highlight the diagnostic dilemma for LIN AST by various methods and their partial inability to correctly assess phenotypic resistance in the presence of a potential underlying LIN resistance mechanism.

## 2. Results

### 2.1. Distribution of LIN Resistance Genes/Mutations among Phenotypically Susceptible Enterococci

From 2019 to 2021, we received a total of 196 *Enterococcus* spp. isolates displaying a linezolid MIC of 4 mg/L when applying the reference method BMD. Isolates were preserved as cryo-cultures shortly after receipt. One isolate could not be re-cultivated from stock and thus was excluded from further analyses.

Of the 195 isolates investigated, species determination confirmed 21 (11%) *E. faecalis* and 174/195 (89%) *E. faecium*. No putative resistance mechanism was detected for 4/21 (19%) *E. faecalis* and 113/174 (65%) *E. faecium* isolates (Table 1). Altogether, 78/195 (40%) of all isolates either contained a putative resistance gene or the G2576T 23S rRNA gene mutation (Table 1). Within *E. faecalis*, one isolate exhibited a 23S rDNA mutation and 16/21 (76%) harbored the gene for the ribosomal protection protein *optrA*. For *E. faecium*, a high proportion [49/174 (28%)] displayed G2576T chromosomal mutations, and, to a lesser extent, a single mobile resistance determinant, such as *poxtA* [7/174 (4%)] or a combination of resistance genes and/or G2576T mutations (Table 1). The gene for the methyltransferase Cfr was detected in two *E. faecium* isolates only (Table 1). It must be noted that we utilized a previously established multiplex-PCR to screen for *cfr, optrA,* and *poxtA* and that some variants thereof might not be identified due to reduced binding of the targeting primers. Thus, the total amount of transferable resistance genes may be underestimated.

### 2.2. Comparison of BMD with Additional AST Methods

#### 2.2.1. Performance of AST Methods on the Set of Isolates with a LIN MIC of 4 mg/L

We carried out additional AST using ETEST^®^ and VITEK2 and investigated the growth of all strains on CHROMagar^TM^ LIN-R plates after 24 h and 48 h of incubation. The MIC results of the different AST methods are displayed in Table 2. Compared to BMD, which is considered the reference method for (LIN) AST in enterococci and which resulted in 100% susceptible isolates (MIC = 4 mg/L) applying EUCAST breakpoints, ETEST^®^ and VITEK2 determined 6.7% and 29%, respectively, as linezolid-resistant (Table 2). Incubation on CHROMagar^TM^ LIN-R resulted in 38% and 42% resistant isolates when evaluated after 24 h and 48 h, respectively, at 37 °C (Table 2).

To estimate the number of false-resistant (major error, ME) and false-susceptible (very major error, VME) isolates as determined by the different AST methods, we made the following assumption: isolates showing none of the known and tested LIN resistance mechanisms (N = 117) were considered genotypically/phenotypically susceptible, whereas those exhibiting either a G2576T 23S rRNA gene mutation, a mobile resistance gene *cfr/optrA/poxtA,* or a combination thereof (N = 78) were considered genotypically/phenotypically resistant.

Due to our study design (only isolates with LIN MIC = 4 mg/L in BMD), ME (false-resistant = genotypically susceptible, but phenotypically resistant) could not be determined for BMD (n.a., not applicable, Table 3). For BMD, VME was 40%, corresponding to isolates that were diagnosed as susceptible but with a genotypic profile capable of mediating phenotypic resistance.

Compared to BMD, the percentage of false-susceptible (= VME) isolates gradually declined from 33% for ETEST^®^ and 13% for VITEK2 to 2.1% for CHROMagar^TM^ LIN-R (48 h of incubation) (Table 3). In contrast, false-resistant (= ME) isolates increased from 0% using ETEST^®^ and 2.1% by VITEK2 to 3.1% and 4.1% when streaking on CHROMagar^TM^ LIN-R agar plates (24 h and 48 h incubation, respectively) (Table 3).

#### 2.2.2. Predictive Ability of Different AST Methods for Genotype-Phenotype Correlation

As outlined above, different AST methods varied with respect to their overall performance. Thus, we further investigated whether these methods exhibit tendencies for the prediction of phenotypic resistance based on their specific genotypic profile. We selected all isolates that either harbored a single resistance gene or the 23S rRNA gene mutation G2576T (*n* = 75) to estimate the positive predictive value (PPV). Isolates with multiple resistance genes and/or chromosomal mutations were excluded due to the inability to differentiate their relative contribution to phenotypic resistance.

The different AST methods varied greatly in their predictive ability according to the underlying potential resistance mechanism (Table 4). Among the LIN resistance genes, ETEST^®^ and VITEK2 showed the highest PPV for *optrA*-positive isolates, 58.8% (10/17) and 11.8% (2/17), respectively, whereas CHROMagar^TM^ LIN-R majorly detected *poxtA*-positive isolates (100%, 7/10) (Table 4).

ETEST^®^ failed to predict phenotypic resistance among *poxtA*-positive strains (0/7, 0%). With only one isolate available, *cfr*-based genotypical resistance demonstrated phenotypic resistant results, and thus a high PPV (100%), for VITEK2 and CHROMagar^TM^ LIN-R, the latter only after 48 h of incubation. With respect to the G2576T chromosomal mutations, ETEST^®^ and VITEK2 demonstrated the highest PPV as compared to gene-mediated resistance (except for *cfr*). For example, VITEK^®^ correctly assessed 72% of G2576T-haboring isolates as resistant as compared to 58.8% and 42.9% for *optrA* and *poxtA*, respectively. In contrast, CHROMagar^TM^ LIN-R generally demonstrated a lower predictive value for the G2576T 23S rRNA gene mutations, especially at 24 h of incubation, when compared to gene-based LIN resistance (86% G2576T vs. 94.1% and 100% for *optrA*- and *poxtA*-positive isolates) (Table 4).

### 2.3. LIN Selection Experiments

**Figure 1:**
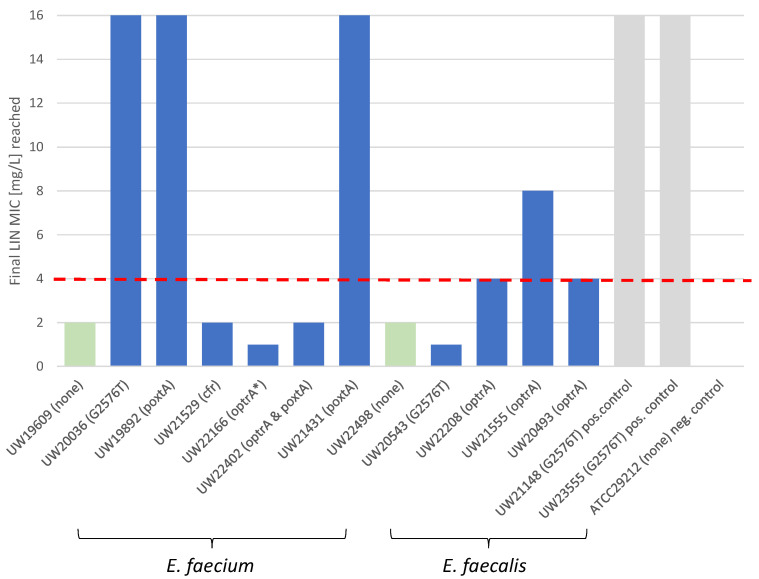
Final MIC of *Enterococcus* spp. isolates after exposure to increasing LIN concentrations. Selected isolates with G2576T ribosomal RNA gene mutations or linezolid resistance genes (blue bars) and without (green bars) were challenged with increasing concentrations of LIN from 0.5 mg/L to 16 mg/L. A total of four isolates reached LIN MICs above the EUCAST clinical breakpoint (>4 mg/L, red dotted line). Two LIN-resistant (*E. faecium* UW21148 and *E. faecalis* UW23555) and one LIN-susceptible isolate (*E. faecalis* ATCC29212) from the NRC strain collection served as positive and negative controls, respectively (grey bars). LIN MIC was determined by broth microdilution. Abbreviations: * isolate which had lost the *optrA* gene during the course of the selection experiments; pos., positive; neg., negative.

We hypothesized, that isolates with a false-susceptible result, hence being genotypically resistant, could potentially adapt to linezolid selective pressure more rapidly. To investigate this, we carried out in vitro selection experiments by gradually increasing LIN concentrations. We selected seven *E. faecium* and five *E. faecalis* with (*n* = 10) and w/o (*n* = 2) G2576T 23S rRNA gene mutations or mobile resistance determinants (Supplementary Table S1). As we have not detected *cfr*- or *poxtA*-positive *E. faecalis* in our study (Table 1), no such isolate could be included. We also excluded combinations of chromosomal mutations and resistance genes, given the generally low number of isolates identified in our study and assuming that frequent recombination of mutated 23S rDNA alleles could shadow the impact of resistant gene expression. After six steps of LIN challenge from sub-inhibitory concentrations of 0.5 mg/L to 16 mg/L, 3/10 of the isolates containing a putative resistance trait had reached MICs above the clinical breakpoint (EUCAST > 4 mg/L), and one isolate (*E. faecalis* UW21555 *optrA*-positive) presented with 4 mg/L and 8 mg/L in the two independent selection experiments (Figure 1 and Supplementary Table S1).

The two strains without a resistance trait as identified in our study (*E. faecium* UW19609 and *E. faecalis* UW22498) and the susceptible negative control *E. faecalis* ATCC29212 were not able to adapt and remained with MIC values below the threshold at the end of the selection experiments (Figure 1). With the sole exception of one *optrA*-positive *E. faecalis* (UW21555, final LIN MIC 4 mg/L and 8 mg/L), no other selected *E. faecalis* was able to adapt to increasing concentrations of LIN (Figure 1). In contrast, the two *poxtA*-positive *E. faecium* (UW19892, UW21431) and an *E. faecium* harboring the G2576T 23S rRNA gene mutation (UW20036) reached MICs of 16 mg/L (Figure 1, Supplementary Table S1). *E. faecium* with *cfr, optrA,* or a combination of *optrA* and *poxtA* were not able to adapt to LIN MIC above 4 mg/L (Figure 1, Supplementary Table S1). In order to rule out that this lack of adaptation is due to the loss of the respective resistance determinant, we examined the presence of the initial resistance trait after LIN selective pressure for all isolates. In one instance, a previously *optrA*-positive *E. faecium* (UW22166, Figure 1, Supplementary Table S1) had lost the resistance gene during the course of both independent experiments. Mutations in the 23S rRNA gene did not revert, as assessed by NheI-restriction experiments, or were detected in previously G2576T mutation-negative isolates.

## 3. Discussion

Linezolid resistance in vancomycin-susceptible, and more importantly, in vancomycin-resistant enterococci, is considered a threat to public health due to limited available treatment options. Therefore, it is important to correctly asses the resistance potential of *Enterococcus* spp. in diagnostic laboratories. We herein examined a set of phenotypically LIN-susceptible isolates (ECUAST susceptible = LIN MIC = 4 mg/L) as determined by the reference method BMD and observed that: (i) 40% of those isolates harbored potential resistance genes or G2576T ribosomal gene mutations, (ii) different AST methods displayed varying results concerning the number of false-susceptible and false-resistant isolates detected, (iii) these methods varied in their ability to predict phenotypic resistances based on the isolates’ genotypical profile, and (iv) “silent” resistance genes could potentially trigger rapid adaptation to LIN under selective pressure.

BMD is considered the reference method for AST of rapidly growing aerobic bacteria [12]; however, assessing resistance to LIN in BMD is a challenging task due to the phenomenon of “trailing growth”. This is defined as the fading of growth over two to three wells [12]. Hence, manual read-out is not only influenced by the growth phenomenon of *Enterococcus* spp. per se, but also by individual visual inspection of the wells, resulting in subjective classification of isolates as resistant or susceptible. As a limitation of our study, our data set only constituted isolates with a LIN MIC of 4 mg/L as determined by BMD and thus, might be prone to a certain degree of measurement bias.

The performance and impact of different AST methods to identify LRE have been assessed previously [13,14,15,16,17]. We found a high proportion (40%) of VME (false-susceptible) for BMD and gradually declining VMEs of 33% for ETEST^®^, 13% for VITEK2, and <5% for CHROMagar^TM^ LIN-R plates. MEs (false-resistant) were assessed as 0% for ETEST^®^, 2.1% for VITEK2, and 4.1% for CHROMagar^TM^ LIN-R (48 h of incubation). A study by Dejoies et al. [13] yielded comparable estimates when applying EUCAST breakpoints with rates of 2.1% ME for VITEK2 and 2.1% ME for ETEST^®^; but in contrast, their VME results showed higher rates of 38.5% for VITEK2 and 23.1% for ETEST^®^. It must be noted that the error rates reported by our colleagues were obtained by using BMD results (susceptible or resistant) as the reference group without considering potential genotypic resistance profiles. This is an important piece of information, implying that reported VME and ME rates for the various methods and subsequent comparisons of the error calculations must be taken with caution, given the different clinical breakpoints applied (EUCAST or CLSI) and the definition of the reference group used.

We categorized our isolates according to EUCAST guidelines for which a LIN MIC = 4 mg/L is considered susceptible, an MIC value that is considered “intermediate” when applying CLSI criteria. Under our study assumption of a genotype–phenotype correlation, the error calculation of VME and ME was substantially different when using CLSI breakpoints, resulting in no VME for BMD (compared to 40% for EUCAST breakpoints), 21% for ETEST^®^ (33% EUCAST), and 7.2% for VITEK2 (13% EUCAST). It seems reasonable to consider CLSI prior to EUCAST for determination of LIN resistance and to flag isolates with an MIC of 4 mg/L, hence drawing the attention of clinicians to a possibly untreatable isolate. With the potential for LIN resistance development under LIN selective pressure for those isolates harboring potential resistance determinants, as seen in our study, the discussion must be continued following further investigations.

In our study, we made a strong assumption that genotypically resistant isolates would correlate with a resistant phenotype. To further investigate whether AST methods differ in their potential to detect the respective underlying resistance trait, we assessed their predictive ability for phenotypic resistance based on the genotypic profile. We observed variability to obtain a resistant phenotype; for example, ETEST^®^ and VITEK2 displayed a much higher PPV for *optrA*-positive isolates compared to *poxtA*. Comparable results were obtained by Dejoies et al., in which the majority of *optrA*-positive isolates reached MIC values above the clinical breakpoint (EUCAST and CLSI) compared to isolates harboring *poxtA* [13]. Furthermore, the prediction of mobile resistance gene determinants by ETEST^®^ and VITEK2 was outperformed by the prediction of phenotypic resistance mediated by G2576T 23S rRNA gene mutation, reaching PPVs as high as 72% (VITEK2). This is somewhat expected as it has been demonstrated that mutation of a single 23R rDNA allele in *E. faecium* is sufficient to elevate MICs above the EUCAST clinical breakpoint [18]. In contrast to VITEK2 and ETEST^®^, CHROMagar^TM^ LIN-R demonstrated higher PPVs for gene-containing isolates (*optrA* and *poxtA*) compared to G2576T chromosomal mutations. Considering the small sample size for *cfr-* and *poxtA*-positive isolates as a limitation for generalization, this is still an interesting observation and requires more in-depth investigations.

We would like to emphasize that the total number of gene-positive isolates may be underestimated and thus affect our estimates for VME, ME, and PPV due to the possible reduced binding of our screening primers to target gene variants. It should also be mentioned that our genotypic assessment focuses on the most frequent 23S rDNA mutation and resistance-mediating transferable genes. Our analysis does not include less relevant 23S rDNA mutations or ribosomal protein alterations, which may result in reduced LIN susceptibility but not LIN resistance in enterococci [19,20,21,22]. This is a limitation of our study.

It is well known that mobile LIN resistance genes may fail to confer phenotypic resistance in some instances that are not well understood [8,23,24,25,26]. Thus, our VME rates are somewhat overestimated. Nevertheless, it is unclear how LIN silent resistance genes, here defined as gene presence but an absence of phenotypic resistance, will respond to LIN selective pressure under LIN therapy. We hypothesized that these silent resistance genes or mutations could potentially allow rapid adaption and resistance development under LIN selective pressure. Our selection experiments confirmed this assumption for *E. faecium* with a G2576T mutation or containing the *poxtA* gene and for one *optrA*-positive *E. faecalis*. However, in some instances, resistance development was not achieved. The results are difficult to interpret at this stage, as the mutation persisted or genes were consistently present (with one exception) over the course of the independent experiments. For *E. faecalis* it was demonstrated that two out of four allelic copies mutated could result in an MIC of 4 mg/L [9], and thus our observation of the G2576T-harboring *E. faecalis* that remained below the resistance threshold might be due to the low number of mutated rRNA alleles that did not increase during LIN selection in vitro. Of note, our results were obtained under laboratory conditions that can only approximate but do not reflect in vivo situations of prolonged LIN therapy. LIN treatment, which is prescribed in concentrations above the sub-inhibitory levels used in our selection experiments, was shown to be associated with an increased risk of developing LIN resistance in *E. faecium* [27]. Moreover, a hospital-wide increasing number of LRE were linked with increased LIN consumption [28]. Thus, failure to develop resistance in 7/10 of our selected isolates investigated might reflect the laboratory conditions and cannot be generalized to a LIN-treated human host.

Relying on phenotypic resistance determination is insufficient to provide the full picture. As seen in our study, 40% of all isolates contained a genotypic resistance marker but were susceptible in BMD (EUCAST breakpoints). It is widely accepted that gene-associated resistance development is highly dependent on the promotor structure, plasmid copy number, or regulation of gene expression [29,30,31], which was not further examined in our study. Silent resistance genes are not unique to enterococci and have been widely reported across the bacteria phylum (reviewed by [32]). However, they can become an unprecedented threat due to the silent transmission of resistance determinants in the hospital environment, as reported for *bla*_OXA-23_-carrying, but imipenem-susceptible *Acinetobacter baumanii* [33]. Furthermore, the transfer of silent genes into a new host may activate the respective resistance trait, as seen for a silent *aadA* gene being expressed after transfer from *E. coli* to *Hafnia alvei* [34]. Imperceptible spreading of LIN silent resistance genes combined with insufficient phenotypic assessment may thus lead to the prescription of non-functional therapeutics, exposing patients to an unnecessary risk for a severe disease outcome. We, therefore, want to emphasize the importance of a combined assessment of genotypic and phenotypic resistance, especially for invasive isolates presenting with a LIN MIC of 4 mg/L. Obtaining the most accurate laboratory results is a prerequisite for the prevention of resistance development or imperceptible resistance gene transmission.

## 4. Materials and Methods

Study material. We included all *Enterococcus* spp. isolates that were sent to the NRC between 2019 and 2021 and demonstrated a linezolid MIC of 4 mg/L using the reference method BMD.

Antimicrobial susceptibility testing. Isolates were cultivated on blood agar followed by BMD using an in-house and accredited procedure and by applying EUCAST clinical breakpoints for resistance determination (EUCAST v11). Here, LIN resistance is defined as MIC > 4 mg/L, whereas an MIC ≤ 4 mg/L is considered susceptible. In comparison, CLSI clinical breakpoints for LIN are as follows: MIC ≤ 2 mg/L “susceptible”, MIC = 4 mg/L “intermediate”, and MIC ≥ 8 mg/L “resistant”. Linezolid insusceptibility was additionally investigated by ETEST^®^ and VITEK2 (bioMérieux, Nuertingen, Germany) according to the manufacturer’s instructions and by cultivation on CHROMagar^TM^ LIN-R (CHROMagar^TM^, Paris, France) for 24 h and 48 h.

Determination of putative resistance mechanism(s). All isolates with an MIC of 4 mg/L in BMD were subjected to DNA extraction and multiplex-PCR in order to screen for *cfr, cfr(B), optrA*, and *poxtA* resistance determinants as described previously [35]. Furthermore, G2576T 23S rRNA gene mutations were determined by an amplification-restriction-based procedure [36].

Data analysis. Results were collectively analyzed using R Studio V4.1.2 and MS Excel Office 2019. For calculating very major error (VME, false-susceptible isolates) and major error (ME, false-resistant isolates), we made the following assumption: the presence of any of the three resistance genes *cfr/optrA/poxtA* and/or the detection of a G2576T mutations could potentially result in phenotypic resistance; thus, these isolates were considered resistant. VME and ME for the different AST methods are expressed as percentage of false-susceptible and false-resistant isolates of all isolates investigated.

Linezolid selection experiments. We included seven *E. faecium* and five *E. faecalis* either harboring a G2576T ribosomal mutation OR a putative resistance gene (*cfr, optrA, poxtA*) OR none of the above (negative control), and two positive controls with LIN MICs > 16 mg/L from the NRC strain collection (one *E. faecium*, one *E. faecalis*), both positive for the G2576T mutation and negative for transferable resistance genes as determined by our inhouse multiplex-PCR (Supplementary Table S1). As for our routine BMD, strains were cultivated first for 3 h in nutrient broth and diluted 1:50 in 0.85% sodium chloride thereafter. Of this solution, 20 µL was used to inoculate a 96-well plate, each well containing 180 µL BHI (1st experiment) or MH broth (2nd experiment) and the respective LIN concentration (see in the following). At step 1, we used sub-inhibitory concentrations of LIN = 0.5 mg/L and LIN = 1 mg/L. Technical duplicates were used throughout. Plates were incubated overnight at 37 °C and growth was visually inspected after 24 h. If growth was detected at the highest concentration (step 1 = 1 mg/L), 20 µL of one of the duplicated cells was transferred to a new 96-well selective plate (step 2). At step 2, we increased the selective pressure by 2-fold but also included the highest concentration from step 1 for recovery (concentrations at step 2 equal 1 mg/L and 2 mg/L, respectively). A growth control without LIN selective pressure was carried along. This procedure was followed for 6 days until LIN = 16 mg/L was reached as the highest concentration. Strains that stopped growing at a certain concentration were stored at −20 °C in 50% glycerol using the previous concentration/well where growth was detected. To assess the final LIN MIC reached, a standard BMD assay was carried out with LIN concentrations ranging from 0.125–32 mg/L. The entire selection experiment was repeated one more time.

## 5. Conclusions

Linezolid resistance genes and mutations can be observed in phenotypically linezolid-susceptible enterococcal isolates suggesting a certain degree of under-representation of their prevalence in clinical isolates. Different antimicrobial susceptibility testing methods vary in their ability to correctly identify putative resistant isolates based on their genotypic resistance profile. In our data set, the reference method, broth microdilution, revealed a substantial number of *Enterococcus* spp. isolates with a LIN MIC of 4 mg/L (= susceptible when applying EUCAST breakpoints) despite the presence of LIN resistance mutations and/or genes. According to CLSI, isolates with a LIN MIC of 4 mg/L are categorized as “intermediate”, which may better indicate a high degree of uncertainty in the classification of these enterococcal isolates. The widespread prevalence of 23S rDNA linezolid resistance mutations and/or possession of LIN mobile resistance determinants in phenotypically susceptible enterococcal isolates may promote the imperceptible spread and trigger a rapid development towards resistance under LIN selective pressure potentially leading to treatment failure.

## Figures and Tables

**Table 1:** Distribution of acquired resistance genes *cfr, optrA, poxtA,* and of G2576T 23S rRNA gene mutations in *E. faecium* and *E. faecalis* isolates displaying a LIN MIC = 4 mg/L (in BMD) collected at the NRC, 2019–2021.

*E. faecium* (N = 174)	*cfr*	*optrA*	*poxtA*	23S rDNA G2576T	*n*	%
	-	-	-	-	**113**	**64.9**
	-	-	-	+	**49**	**28.2**
	-	-	+	-	**7**	**4.0**
	-	+	-	-	**1**	**0.6**
	-	+	-	+	**1**	**0.6**
	-	+	+	-	**1**	**0.6**
	+	-	-	-	**1**	**0.6**
	+	-	-	+	**1**	**0.6**
total					**174**	**100**
***E. faecalis*** **(N = 21)**	***cfr***	***optrA***	***poxtA***	**23S rDNA** **G2576T**	***n***	**%**
	-	+	-	-	**16**	**76.0**
	-	-	-	-	**4**	**19.0**
	-	-	-	+	**1**	**5.0**
total					**21**	**100**

**Table 2:** Linezolid resistance determination by different AST methods among the set of isolates with a LIN MIC of 4 mg/L (in BMD) collected at the NRC, 2019–2021.

LIN MIC	BMD		ETEST^®^		VITEK2		CHROMagar^TM^ LIN-R 24 h ^1^		CHROMagar^TM^ LIN-R 48 h ^1^	
	*n*	%	*n*	%	*n*	%	*n*	%	*n*	%
0.75	-		13	6.7%	-					
1	-		44	23%	4	2.1%				
1.5	-		35	18%	-					
2	-		33	17%	121	62%				
3	-		31	16%	-					
4	195	100%	26	13%	13	6.7%	120	62%	113	58%
>4	-		13	6.7%	57	29%	75	38%	82	42%

^1^ no MIC can be inferred from CHROMagar^TM^ LIN-R plates, therefore, growth on plates was considered resistant (>4 mg/L), and no growth as susceptible (=4 mg/L). “-”, no data available for the respective concentrations (BMD, VITEK2).

**Table 3:** Major error and very major error of different AST methods to correctly identify linezolid-resistant and linezolid-susceptible *Enterococcus* spp. isolates among the set of isolates with a LIN MIC of 4 mg/L (in BMD) collected at the NRC, 2019–2021.

	BMD		ETEST^®^		VITEK2		CHROMagar^TM^ LIN-R 24 h ^1^		CHROMagar^TM^ LIN-R 48 h ^1^	
	*n*	%	*n*	%	*n*	%	*n*	%	*n*	%
ME ^1^	n.a.		0	0%	4	2.1%	6	3.1%	8	4.1%
VME ^2^	78	40%	65	33%	25	13%	9	4.6%	4	2.1%

^1^ Major error (false-resistant, phenotypically resistant but genotypically susceptible isolates); ^2^ Very major error (false-susceptible, phenotypically susceptible but genotypically resistant isolates). Strains containing either a G2576T ribosomal mutation and/or a resistance gene (*cfr, optrA, poxtA*) were considered genotypically resistant. False-resistant and false-susceptible isolates were defined under the study assumption that genotypically resistant isolates, with G2576T mutations or transferable resistance genes, should result in a resistant phenotype. Abbreviations: n.a., not applicable (for BMD, no phenotypically resistant isolates were included in the study).

**Table 4:** PPV of different AST methods for genotype–phenotype correlation according to the potential LIN resistance trait of the study isolates with a LIN MIC of 4 mg/L (in BMD) collected at the NRC, 2019–2021.

AST Method	AST Result	*cfr*		*optrA*		*poxtA*		G2576T	
		*n*	PPV%	*n*	PPV%	*n*	PPV%	*n*	PPV%
ETEST^®^	Susceptible	1	0%	15	11.8%	7	0%	39	22%
	Resistant	0		2		0		11	
VITEK2	Susceptible	0	100%	7	58.8%	4	42.9%	36	72%
	Resistant	1		10		3		14	
CHROMagar^TM^ LIN-R 24 h	Susceptible	1	0%	1	94.1%	0	100%	7	86%
	Resistant	0		16		7		43	
CHROMagar^TM^ LIN-R 48 h	Susceptible	0	100%	1	94.1%	0	100%	3	94%
	Resistant	1		16		7		47	

## Data Availability

The data presented in this study are available on request from the corresponding author.

## Supplementary Materials

The following are available online at https://www.mdpi.com/article/10.3390/antibiotics13010101/s1, Table S1: *E. faecium* (*n* = 7) and *E. faecalis* (*n* = 5) isolates for LIN selective pressure experiments from the study set of LIN MIC = 4 mg/L (in BMD) strains collected by the NRC, 2019–2021.
