# Supplementary material for: Linezolid Resistance Genes and Mutations among Linezolid-Susceptible Enterococcus spp.—A Loose Cannon?
Source: Antibiotics (Basel). 2024 Jan 19;13(1):101. doi: 10.3390/antibiotics13010101 (PMC10812394; doi:10.3390/antibiotics13010101)
Supplement: Supplementary file 1 [file antibiotics-13-00101-s001.zip › antibiotics-2775892-supplementary_revised-1.pdf]

Supplementary material

Table S1. *E. faecium* (n=7) and *E. faecalis* (n=5) isolates used in LIN selective pressure experiments and selected from isolates with a LIN MIC of 4mg/L (in BMD) collected at the NRC, 2019-2021.

Isolate	Species	LIN resistance mutation or LIN resistance gene content	Final LIN MIC [mg/L], experiment 1[1]	Final LIN MIC [mg/L], experiment 2[1]
**UW19609**	*E. faecium*	None	2	2
**UW20036**	*E. faecium*	G2576T	16	16
**UW19892**	*E. faecium*	*poxtA*	8	16
**UW21529**	*E. faecium*	*cfr*	2	2
**UW22166**	*E. faecium*	*optrA*	1[2]	1[2]
**UW22402**	*E. faecium*	*optrA* & *poxtA*	2	2
**UW21431**	*E. faecium*	*poxtA*	16	16
**UW22498**	*E. faecalis*	None	4	2
**UW20543**	*E. faecalis*	G2576T	2	1
**UW22208**	*E. faecalis*	*optrA*	2	4
**UW21555**	*E. faecalis*	*optrA*	4	8
**UW20493**	*E. faecalis*	*optrA*	4	4
**UW21148**	*E. faecium*	G2576T; positive control from NRC strain collection	16	16
**UW23555**	*E. faecalis*	G2576T; positive control from NRC strain collection	32	16
**ATCC29212**	*E. faecalis*	None; negative control from NRC strain collection	0.5	0.5

[1]LIN MIC assessed by broth microdilution method; [2]isolate that had lost the resistance gene after LIN selective pressure
